# The elements of end-of-life care provision in paediatric intensive care units: a systematic integrative review

**DOI:** 10.1186/s12904-024-01512-5

**Published:** 2024-07-25

**Authors:** Fanny Adistie, Susan Neilson, Karen L. Shaw, Betul Bay, Nikolaos Efstathiou

**Affiliations:** 1 School of Nursing and Midwifery, College of Medical and Dental Sciences, University of Birmingham, Birmingham, UK; 2 Faculty of Nursing, Universitas Padjadjaran, Bandung, Indonesia; 3 Institute of Applied Health Research, University of Birmingham, Birmingham, UK; 4 School of Nursing, Faculty of Health Sciences, University of Ottawa, Ottawa, Canada

**Keywords:** End-of-life care, Integrative review, Palliative care, Paediatric intensive care unit

## Abstract

**Background:**

Deaths in paediatric intensive care units (PICUs) are not uncommon. End-of-life care in PICUs is generally considered more challenging than other settings since it is framed within a context where care is focused on curative or life-sustaining treatments for children who are seriously ill. This review aimed to identify and synthesise literature related to the essential elements in the provision of end-of-life care in the PICU from the perspectives of both healthcare professionals (HCPs) and families.

**Methods:**

A systematic integrative review was conducted by searching EMBASE, CINAHL, MEDLINE, Nursing and Allied Health Database, PsycINFO, Scopus, Web of Science, and Google Scholar databases. Grey literature was searched via Electronic Theses Online Service (EthOS), OpenGrey, Grey literature report. Additionally, hand searches were performed by checking the reference lists of all included papers. Inclusion and exclusion criteria were used to screen retrieved papers by two reviewers independently. The findings were analysed using a constant comparative method.

**Results:**

Twenty-one studies met the inclusion criteria. Three elements in end-of-life care provision for children in the PICUs were identified: 1) Assessment of entering the end-of-life stage; 2) Discussion with parents and decision making; 3) End of life care processes, including care provided during the dying phase, care provided at the time of death, and care provided after death.

**Conclusion:**

The focus of end-of-life care in PICUs varies depending on HCPs’ and families’ preferences, at different stages such as during the dying phase, at the time of death, and after the child died. Tailoring end-of-life care to families’ beliefs and rituals was acknowledged as important by PICU HCPs. This review also emphasises the importance of HCPs collaborating to provide the optimum end-of-life care in the PICU and involving a palliative care team in end-of-life care.

## Background

Although childhood death is a rare phenomenon, when it occurs either at home or in a healthcare setting, it can be a traumatic event [1, 2]. A substantial number of these deaths will occur in hospitals and more specifically in paediatric intensive care units (PICUs) [3]. Mortality rates in PICUs vary across the world, with developed countries reporting very low rates; for example, in the United Kingdom (UK) and the Republic of Ireland, the mortality rate among children admitted to PICUs in 2021 was 3.3% [4] and in Sweden during the period 2008–2016 the mortality rate was 2.93% [5]. Mortality rates in middle-income countries’ PICUs are higher, for example a study in the PICU of the main referral paediatric hospital in Iran reported a mortality rate of 12.2% for 2020 [6], and a study in the PICU of a tertiary hospital in Indonesia reported a mortality rate of 10.7% for 2018 [7]. While some deaths in PICUs occur within minutes to days, others can occur days to years after multiple PICU admissions, primarily because access to technologically advanced life-sustaining treatments alters disease trajectories and contributes to chronic medical complexities [8].

The importance of high-quality end-of-life care for these children and families has been emphasised, however its provision is acknowledged as challenging globally. For example, children with life-threatening conditions and their families in the UK encounter many social, economic, and cultural barriers when trying to access care, including the complex needs of families facing the death of a child [9]. In addition, healthcare professionals (HCPs) may feel unprepared to provide compassionate and sensitive care at the end of life [10, 11]. A small survey of 10 parents in India reported that parental caring for dying children with cancer created a sense of failure, powerlessness, and guilt [12]. In the same study, 40% of the parents reported financial concerns contributed to their distress during the child’s terminal illness and only 40% rated emotional support by HCPs as excellent [12]. For HCPs, providing care for children as well as for their families in a palliative situation, is a highly demanding and stressful task, particularly when the child’s health deteriorates, and transitions to end-of-life care [13].

End-of-life care in PICUs is generally considered more challenging than other settings, because of the focus on curative or life-sustaining treatments for children who are seriously ill. Hence, end-of-life care may not be considered until very late [14]. In addition, parents may have difficulty with the decision-making process related to withdrawing treatment from a child, significantly impacting their psychological well-being [15]. Therefore, it is indisputable that there are significant challenges in the provision of palliative and end-of-life care in the PICUs.

Considering the challenges faced by all involved in palliative and end-of-life care in PICUs, it is not surprising that considerable research has been undertaken over the years with HCPs and families to explore experiences, perspectives, attitudes and impact of end-of-life care [16]. Three reviews have been published between 2015–2022, specifically related to end-of-life care in PICUs. The most recent review aimed to synthesise the experiences of parents who endured the death of their child in the PICU and what end-of-life care they perceived as supportive [17]. This review found that parents need to be able to effectively interact with HCPs to fulfil their parental responsibilities and participate in important decisions regarding treatment for their child. The review by Mu et al. [18] synthesised evidence around nurses’ experiences of end-of-life care in PICUs mentioning that nurses experienced inadequate communication, emotional strain and moral distress resulting from medical futility. Finally, Howes [19] reviewed the provision of end-of-life care in PICU and the options available to children and families. The authors found parents’ discomfort with withdrawing their child’s ventilation, inadequate communication, and limited accessibility to children’s hospices, had to be overcome before transferring the children out of PICU and continuing end-of-life care. While these reviews provide important insights, there is a need to update the knowledge base. This is because the majority of existing reviews considered health professionals’ or families’ findings separately, while the only review that considered both, included evidence up to 2013. It is imperative to undertake an up to date and more comprehensive review by including various research designs and grey literature to analyse and synthesise findings from the perspectives of family and HCPs. Thus, this review aims to synthesise evidence to identify the essential elements in the provision of end-of-life care in the PICU from the parents’ and HCPs’ perspective.

## Methods

### Study design

This integrative review was conducted using Whittemore and Knafl [20] methodology which consists of five steps: identification of the problem, literature search, data evaluation, data analysis, and presentation. The strength of integrative review is its capacity to analyse research literature, evaluate evidence quality, merge findings from various research designs, generate research questions, and develop an excellent empirical foundation that promotes evidence-based practice [21, 22]. The integrative review methodology allowed for a holistic understanding of PICU palliative care, which would not be achieved by other review methodologies such as systematic or scoping review [23]. This review was registered on PROSPERO (CRD42022346518). In the absence of a specific integrative review reporting guide, the Preferred Reporting Items for Systematic Reviews and Meta-Analyses (PRISMA) checklist has been used to report this review [24].

### Identification of the problem

To identify the problem and formulate the review question, the template population, intervention/interest, comparison/context, outcome, time, and study design (PICOTS) was used (Table 1) [21]. The questions in this review were ‘What does end-of-life care involve in PICUs?’, there were two sub questions: (1) Who provides end-of-life care in the PICU? and (2) How do healthcare professionals provide end-of-life care in the PICU?

**Table 1:** PICOTS elements

**Population**	Paediatric patients in the end-of-life stage or have died in PICU, their family, and healthcare professionals
Intervention/interest	Provision of end-of-life care
Comparison/context	No comparator Paediatric intensive care unit context
Outcomes	High quality of end-of-life care provision in PICU
Time	Studies published after January 2014
Study design	Quantitative, qualitative, and mixed method studies

### Eligibility criteria

#### Inclusion criteria

Primary quantitative, qualitative, and mixed method studies, focused on end-of-life care in PICUs were included in the review. Studies were included if the population were HCPs and/or family who had experienced the death of a child in PICU. Studies with data about paediatric patients receiving end-of-life care in PICU were included since they provide indirectly perspectives of HCPs and/or family. Studies published from January 2014 were eligible for inclusion, as a previous review on the provision of end-of-life care in PICU [19] is available with literature included up to 2013. Only studies reported in English language were included.

#### Exclusion criteria

Secondary research in the form of systematic reviews or any other type of reviews were excluded. Articles concerning practices relating to neonatal and adult Intensive Care Unit (ICU) were not considered. However, studies conducted in Neonatal Intensive Care Unit (NICU) and PICU settings were included if the authors presented separately findings related to the PICU only. A full list of the inclusion and exclusion criteria is presented in Table 2.

**Table 2:** Inclusion and exclusion criteria

Inclusion	Exclusion
Participant on the research were: - Health care professionals - Family Papers including data about paediatric patients receiving end-of-life care in PICU	Papers solely focused on the paediatric patient who experienced sudden death
The setting of the study was in PICU	Adult ICU NICU
Research in English in the time span of January 2014 until April 2023 in the form of: - Quantitative studies - Qualitative studies - Mixed-method studies - Case studies - Theses/Dissertations - Conference articles	- Conference abstracts - Opinion papers - Editorials - Reviews - Simulation studies - Pilot studies - Instrument development and assessment

### Search strategy

The following electronic databases were searched to ensure that relevant literature was captured: EMBASE, CINAHL, Medline, Nursing and Allied Health Database, PsycINFO, Scopus, Web of Science, and Google Scholar. Grey literature was searched via Electronic Theses Online Service (EthOS), OpenGrey, Grey literature report. The last search was run on April 3rd, 2023.

The keywords were developed in consultation with a subject specific librarian at the University of Birmingham. Medical Subject Heading terms and any relevant terminology and truncation symbol (*), and Boolean operators AND, OR, NOT were used during database searches. The following terms were used in the search strategy “health care professionals” OR “health care staff” OR “nurses” OR “doctors” OR “psychologists” OR “pharmacists” OR “chaplain” OR “famil*” OR “parents” AND “end-of-life care” OR “palliative care” OR “terminal care” OR “advance care planning” OR “life support care” OR “dying” OR “EOL” AND “paediatric intensive care unit” OR “pediatric intensive care unit” OR “paediatric critical care unit” OR “pediatric critical care unit” OR “PICU”.

### Selection of studies

The results of the searches in each database were exported and deduplicated in EndNote version 20 [25] and manually checked by hand-searching for remaining duplicates [26]. Following deduplication, all papers were uploaded on Rayyan to facilitate collaboration between reviewers in screening and labelling articles (include/ exclude/ maybe) in blind mode and then compare the label they generated [27]. Two reviewers independently decided the potential eligibility of each study by title and abstract. Due to the volume of retrieved papers the task was divided between reviewers (FA & NE, FA & SN, FA & KLS). Following this level of screening any disagreements were explored and discussed, and if these were not resolved then a third reviewer was consulted [28].

Additional papers were identified during the screening process by checking the reference lists of all included papers, and their abstracts were reviewed in a similar manner. Finally, full text screening was done independently by two reviewers (FA, NE) and the full list of included papers was agreed by all reviewers.

### Data extraction

Data from included papers were extracted by two reviewers (FA, BB) into a Microsoft Excel spreadsheet using a predetermined form which included the authors, year of publication, origin of the research, study aim, designs and data collection and analysis methods, sampling strategy, participants’ characteristics, main findings, financial support, conflict of interest and the strengths and limitations of the study. The review team developed the data extraction sheet which was pilot tested on five studies and minor alterations were made. Following the data extraction, a sample of 25% of the data extraction was double-checked by NE and no discrepancies were found.

### Quality appraisal

Critical appraisal tools by Critical Appraisal Skills Programme (CASP) were used to assess the quality of the papers that met the inclusion criteria, and also to consider their validity, results and relevance to the context [29]. The variety of literature that can be incorporated into an integrative review necessitates a variety of evaluation techniques [30]. Since methodologies in some studies could not be appraised using CASP checklists, the Mixed Methods Appraisal Tool (MMAT) by Hong et al. [31] was also used in this integrative review. The appraisal of the included studies was conducted by FA. A sample of papers (*n* = 8) was appraised independently and validated by NE, SN, and KLS. There was 95% agreement between reviewers on the appraisal checklists of these eight papers. Disagreements were discussed, and final appraisals agreed by all. No selected study was excluded from the review solely based on the quality, as studies with lower quality can still contribute to a review [32].

### Data synthesis

The four phases of constant comparative methods (data reduction, data display, data comparison, and conclusion drawing and verification) proposed by Whittemore and Knafl which provide the most thorough overview of a systematic analytic strategy were used in this review [33, 34]. In the first phase, one reviewer (FA) grouped the findings from the included articles. The quantitative data were transformed into qualitative data (qualitising) identifying what was measured, then categorizing and translating or converting the data into textual descriptions [35]. All findings from the papers were extracted in a spreadsheet and tabulated. In the data comparison phase, the reviewer constantly compared the findings and uncovered patterns, commonalities, and differences that led to the creation of themes. In the last phase, the reviewer (FA) generated the conclusion and verified the themes with the other reviewers.

## Results

**Fig. 1:**
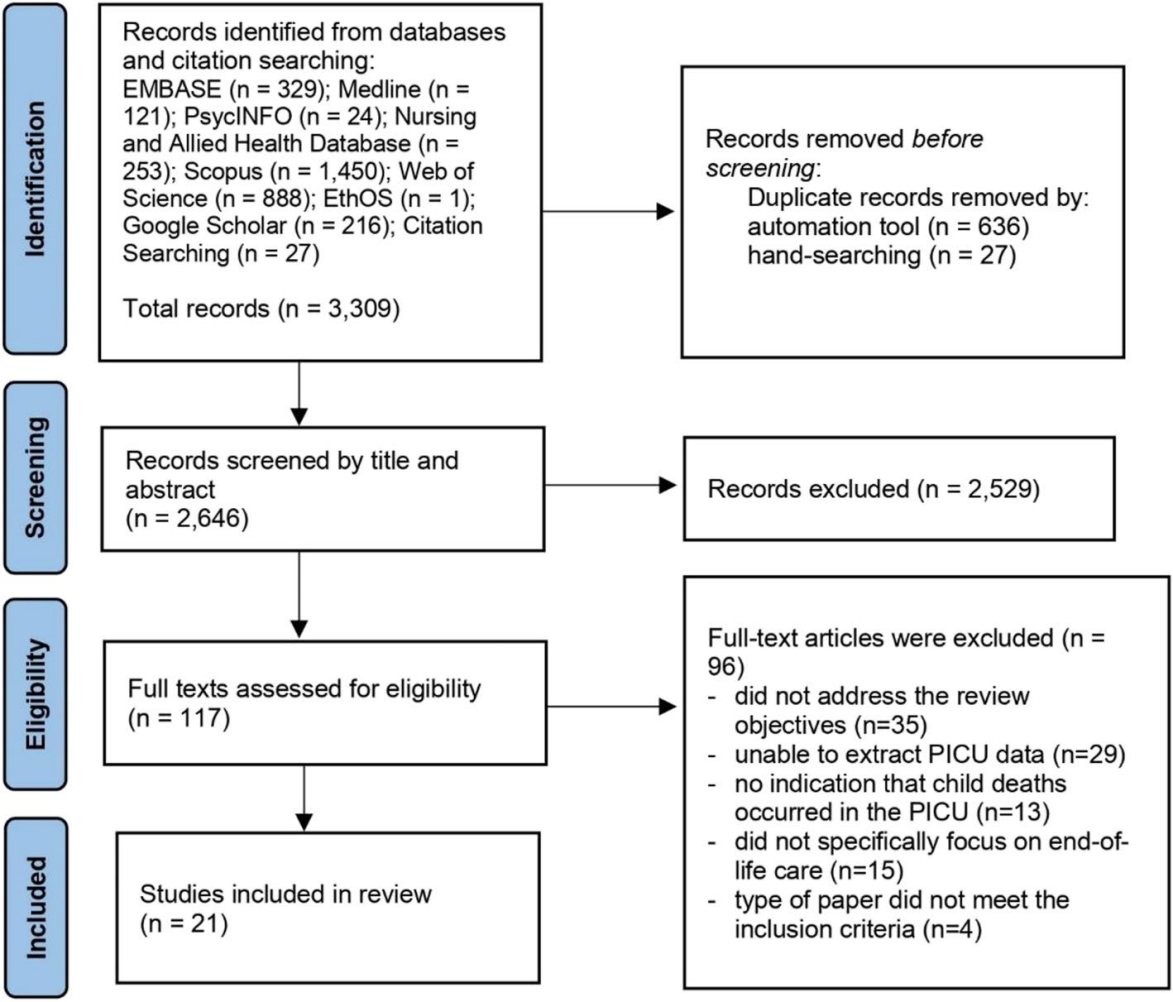
PRISMA flow diagram [24]

A total of 3,309 papers were retrieved from databases and citation searching. After removing duplicates, 2,646 remained. Of these, 2,465 papers were discarded at title/abstract screening level. The remaining 117 papers were examined at full text level. Ninety-six did not meet inclusion criteria, which left 21 papers to be included in the review (Fig. 1).

### Study characteristics

The 21 papers included in this review (Table 3) reported findings from across the globe: 5 (23.8%) from Europe, 5 (23.8%) from Asia, 4 (19%) from South America, 4 (19%) from North America, and 3 (14.3%) from Australia. Twelve (57.1%) articles reported studies that had used a qualitative approach, 7 (33.3%) were quantitative studies, and 2 (9.5%) were mixed-methods studies. In 12 (57.1%) studies, participants were healthcare professionals, in two (9.5%) studies participants were bereaved parents, six (28.6%) studies had paediatric patients as participants, and one (4.7%) study had healthcare professionals and family as participants (Table 4). Most of the included papers (18 out of 21) were of very good quality (meeting 90–100% of the critical appraisal criteria). The remaining papers were of good quality (meeting 71–77% of the critical appraisal criteria). (Table 3, available at the end of the manuscript, and Table 4 around here).

**Table 3:** Summary table of the studies

**Author—Country**	**Aim**	**Study Design/ Data Collection Method/ Data Analysis Method**	**Participants**	**Major Findings**	**Limitations**	**Critical Appraisal Result**
Bloomer et al. [38] – Australia	To describes the nurses’ endeavours to create normality amidst the sadness and grief of the death of a child in paediatric and neonatal ICU	Qualitative study using focused group and individual interviews. Data analysis used inductive content analysis	Twenty-one registered nurses from NICU (*n* = 8) and PICU (*n* = 13)	1) Respecting the child as a person, especially their physical appearance 2) Creating opportunities for family involvement/connection 3) Collecting mementos seemed a common way to connect family members to their infant Participants stressed that any infant/child death is a life cut too short, and thus any way in which they could accommodate the wishes of the family were prioritised	The findings cannot be assumed to be generalisable to all PICU and NICU settings because of undertaking in two hospitals	CASP 10/10
Bloomer et al. [37] – Australia	To explore how NICU/PICU nurses care for families before and after death; to explore the nurses’ perspectives on their preparedness/ability to provide family care; and to determine the emotional content of language used by nurse participants	A Mixed method study with focus group interviews utilised a semi-structured conversational approach. Data were analysed using content analysis. Quantitative data analysed using Mann Whitney and Kruskall–Wallis non-parametric tests	Twenty-two registered nurses from NICU (*n* = 9) and PICU (*n* = 13)	Providing aggressive treatment to a dying child/infant whilst simultaneously caring for the family caused discomfort and frustration for nurses. Nurses sometimes delayed death to allow families to prepare, which enabled differentiation between types of emotional talk such as anger talk, anxiety talk and sadness talk PICU nurses had significantly more anxiety talk than NICU nurses	The collection of demographic data, might have provided further insights in this study	MMAT 13/17
Bobillo-Perez et al. [45]—Spain	To describe how end-of-life care is managed when life-support limitation is decided in a PICU and to analyse the influence of the further development of the Palliative Care Unit	A retrospective study. Data collection method used participant observation with field notes and in-depth interviews. Data analysis used content analysis	175 patients who died in the PICU after the life-support limitation (LSL) decision	1) The primary reason for life-support limitation was the negative progression of the underlying illness 2) In a significant number of cases, families actively participated in the decision-making process 3) The increased presence of a Palliative Care Unit, coupled with the growing assurance among intensivists in administering end-of-life care, may play a crucial role in enhancing the overall quality of end-of-life care provided	1) The study’s findings are based on retrospective accounts 2) Single-center design are limitations, although this confers homogeneity to the study 3) The analysis of the impact of the Palliative Care Unit in a hospital is not complete without considering those patients who are managed in a general hospital ward or at home	MMAT 7/7
Broden et al. [55] – US	To examine associations between patient characteristics, circumstances of death, and nursing care requirements for children who died in the PICU	A secondary analysis of the data set from the Randomised Evaluation of Sedation Titration/The Face, Legs, Activity, Cry, and Consolability (FLACC) scale, Individualised Numeric Rating Scale, or Wong-Baker Faces scale, the State Behavioral Scale, the Nine Equivalents of Nursing Manpower Use Score (NEMS). Kruskal–Wallis tests for continuous variables and Fisher exact tests for categorical variables	his analysis included 104 children; 67 died after withdrawal of life-sustaining treatments; 21, after failed resuscitation; and 16, after brain death	1) Daily pain and sedation scores indicated patients’ comfort was well managed 2) Patients with longer PICU stays more often experienced pain and agitation on the day of death 3) Illness trajectory was associated with pain scores 4) Specifically, children with cancer had higher pain scores than children with acute illness trajectories 5) Patients with cancer and longer PICU stays had more episodic pain and agitation and may require increased attention to comfort management 6) Decisions to withdraw LST may occur in the context of quickly evolving clinical scenarios, with all options pursued until the very final moments preceding a child’s death	1) Limited level of detail about nursing care at EOL in the PICU 2) This secondary analysis is subject to the restrictions of the parent study, which may limit the generalisability of their findings	MMAT 7/7
Butler et al. [36] – Australia	To explores early and ongoing relationships between parents and health care staff when a child dies in intensive care	Constructivist grounded theory with semi-structured interviews/ The constant comparative methods and theoretical memoing	Twenty-six parents (18 mothers and eight fathers) from four Australian PICUs participated in the study	1) Saying goodbye Commonly, parents did not know how to say goodbye to their child. Health care providers adopted a dominant role, guiding the parents through the process of farewelling their child 2) Going home The relationship between parents and health care providers again adapted to the parents’ changing needs, focusing on supporting them to leave the hospital without their child 3) Seeking supports Described the parents’ need for longer term ongoing support, typically with someone with whom they had already developed a relationship in the PICU	1) Most of the parents in this study were Caucasian, with limited prior PICU experience 2) The authors were unable to recruit any families of children who were brain dead or who participated in organ donation 3) The sample also included only one family whose child died from unsuccessful CPR. It is possible that these families have differing follow-up needs 4) The lack of data from parents of school-age children may still limit the relevance of the findings to these families 5) The authors also had difficulties with participant recruitment at on study site, resulting in a loss of bereavement follow-up records	CASP 10/10
Furtado et al. [39] – Brazil	To determine the prevalence of life support limitation (LSL) in patients who died after at least 24 h of a paediatric intensive care unit (PICU) stay, parent participation and to describe how this type of care is delivered	Retrospective cohort study/ Medical record/ Pearson’s chi-square test or Fisher’s exact test, the nonparametric Mann–-Whitney U test	Fifty-three patients in the PICU from March 1, 2015, to February 28, 2019	1) The occurrence of LSL report was observed in 45.3% of cases 2) Among the 24 patients with an LSL report in their medical records, only one did not have DNR order in place 3) Half of the patients with an LSL report had their life support withdrawn 4) Patients with an LSL report tended to have longer stayed in the PICU, were older, had a higher rate of parental presence at the time of death, and exhibited greater severity on admission 4) A substantial increase in the prevalence of LSL reports	1) The study used non-validated instruments 2) Lack of consistency in the definitions 3) The use of data that is not always complete 4) The usual absence of objective documentation 5) Recording bias because of reviewing medical records 6) Study was conducted in a single centre	MMAT 7/7
Grunauer et al. [43] – Ecuador	To assess how PICUs around the world implement grief and bereavement care (GBC) as part of an integrated model of care relative to the Initiative for Pediatric Palliative Care (IPPC) curriculum	Mixed method study with cross-sectional, prospective survey study. Qualitative data analysed using content analysis and quantitative data using multilevel generalised linear models (GLM) with a Gaussian distribution	34 PICUs in 18 countries across Asia (15), Latin America (7), North America (5), Europe (5), and Africa (2). High-income countries (HICs): 32.4%, upper middle-income countries (UMICs): 44.1%, low middle-income and low-income countries (LMI/LICs): 23.5%	1) The study found statistically significant differences in GBC fulfilment scores between HICs and UMICs, and between HICs and LMICs 2) PICUs provide some GBC, independent of income, but barriers include lack of financial support, time, and training, overall unit culture, presence of a palliative care consultation service, and varying cultural perceptions of child death 3) Disparities in GBC for families and HCPs exist and were related to the native countries’ income level	1) No information regarding the involved institutions whether they were from urban, suburban, or rural areas and public, private, or public–private funding 2) Authors did not include the variable of the medical professional seniority which may resulting in varied opinions on grief and bereavement care 3) Underrepresentation of practical issues of the service in the countries because of determining GBC fulfilment exclusively via assessment of the IPPC curriculum	MMAT 13/17
Jongaramraung et al. [49] – Thailand	To investigate end-of-life decisions, based on health professionals’ perspectives, for children admitted to the paediatric intensive care unit	Qualitative descriptive study/ In-depth interview/ Thematic analysis	19 participants including two doctors and 17 nurses who were involved in EOL decisions for terminally ill children	1) Doctors were responsible for making a definite EOL diagnosis before commencing the EOL decision for terminally ill children 2) The doctors needed to consult relevant medical specialists to confirm whether critically ill children had truly arrived at the EOL stage or could not be cured 3) In case of misunderstanding and need to making decision, doctors directly explained the child’s status to parents 4) Doctors and nurses respected and accepted parental decisions, even if those decisions changed later 5) Medical management of each EOL option was generally the main role and responsibility of doctors	1) The findings of this study represent the healthcare professionals’ perspectives at a given point in time 2) Recollection might be a potential bias since all participants had to recall relevant events or situations retrospectively 3) As the findings of this study are based on healthcare professionals’ viewpoints, it might not reflect actual parents’ reasons for choosing or changing an EOL option	CASP 9/10
Lewis-Newby et al. [54] – US	Examined the current state of clinician perspective on communication with families of dying children in the PICU	Prospective case series/ Self-administered survey/ Descriptive statistics, Hierarchical linear regression modelling, T-tests, Multivariable regression analysis	Five hundred sixty-five surveys were returned by 287 clinicians for 169 children who died. Of the 565 surveys, 38% were completed by nurses, 36% by doctors, and 26% by psychosocial staff	1) Location of communication: Most communication during the last three days of life occurred at the bedside. Relatively little communication was reported to have occurred over the phone or in nonprivate settings 2) Barriers to end-of-life care: The most frequently reported barrier was ‘‘patient too sick to allow interaction with family’’ 3) Quality of communication: The research site, type of clinician, patient age, gender, and clinical characteristics had no impact on QOC. However, when the dying child was non-white, the QOC was rated lower patients	1) Low response rate 2) In the intervening time since data collection, PICU cultural changes have occurred regarding family-clinician communication, family-centered care, and even changes to physical spaces within PICUs for family communication	MMAT 7/7
Meert et al. [53] – US	To describe variability in end-of-life practices among tertiary care paediatric intensive care units (PICUs) in the U.S	Descriptive study/ A secondary analysis of prospectively collected data/ Pearson chi-square or Fisher’s exact test	275 (2.7%, range across sites 1.3%–5.0%) patients from the primary study sample	1) Mortality rates for infants and children admitted to tertiary care PICUs in the U.S. for the first time during a hospitalisation are low 2) The most common form of limitation of support observed was avoidance of cardiac compressions 3) The most common form of withdrawal of support observed was discontinuation of mechanical ventilation 4) Over two-thirds (68%) of deaths occurred after support was limited or withdrawn, with minimal variability observed across sites 5) Respiratory failure and congenital heart disease were the most common PICU admission diagnoses among patients who died 6) Overall, 7% of patients who died donated organs 7) The percentage that had an autopsy performed (67%) also varied across sites (50%–100%)	1) A lack of information regarding why various end-of-life decisions were made such as limitation or withdrawal of support; and the possibility that some family-clinician discussions about limitation or withdrawal of support were missed during data collection 2) Lack of data to evaluate reasons behind practice variability for specific aspects of end-of-life care 3) The collection of baselines PCPC and FSS scores from historical data, and the inability to evaluate differences across sites for some clinically important variables due to an insufficient number of subjects in some categories 4) Further explore the issue of racial disparities due to missing race and ethnicity data	MMAT 7/7
Mesukko et al. [50] – Thailand	To develop a set of Palliative Care Guidelines for Doctors and Nurses in Paediatric Intensive Care (PCGPHPIC) caring for children and their families in a university hospital in Thailand	A qualitative study using participatory action research (PAR). The data collection processed through three phases consisted of preparation phase, situational analysis phase, and plan development phase. Data were analysed by content analysis	Forty-four healthcare professionals, consisting of doctors, registered nurses, and practical nurses	Five critical components that needed to be included in the Palliative Care Guidelines for Doctors and Nurses in Paediatric Intensive Care: Breaking bad news, Decision making, Care before death, Imminent death care, and Care after death	1) This study was undertaken in a paediatric intensive care unit at one university hospital using PAR 2) The family members who had experience with dying children in PICU were not involved in this study and they would add valuable information for guideline revision in the future	CASP 10/10
Mitchell and Dale [51]- United Kingdom	To explore the experiences of senior medical and nursing staff regarding the challenges related to Advance Care Planning (ACP) in children and young people with life-limiting illnesses in the PICU and opportunities for improvement	Qualitative study with semi-structured, interviews, and thematic content analysis	Eight PICU consultants and six senior nurses	Four main themes emerged from the study: 1) Recognition of an illness as ‘life-limiting’ 2) ACP as a multi-disciplinary, structured process 3) The value of ACP 4) Adverse consequences of inadequate ACP	1) Small number of participants who were brought in via the same PICU 2) Several emerging themes were not reported 3) The conclusions were derived from retrospective narratives that might have undergone modifications over time	CASP 9/10
Mitchell et al. [52] – UK	To give a comprehensive understanding of the experiences and perspectives of bereaved parents who have experienced in end-of-life care decision-making for children with life-limiting or life-threatening conditions in the paediatric intensive care unit (PICU)	A qualitative study using semi-structured interview and thematic analysis	Seventeen parents of 11 children who had died in the PICU	1) Parents have significant knowledge and experiences that influence the decision-making process 2) Trusted relationships with HCPs are key to supporting parents making end of life decisions 3) Verbal and non-verbal communication with HCPs impacts on the family experience 4) Engaging with end-of-life care decision-making can be emotionally overwhelming but becomes possible if parents reach a ‘place of acceptance’ 5) Families perceive benefits to receiving end of life care for their child in a PICU	1) The number of participants is relatively small and recruited through the same PICU 2) The parents who felt unable to participate may have had views, experiences and perceptions that were different 3) The study’s findings are based on retrospective accounts 4) The authors did not capture the experiences and perceptions of families who are currently in the process of making end-of-life care decisions for their children, or the views of any children or young people regarding their own end of life care decision-making	CASP 9/10
Mutair et al. [44] – Saudi Arabia	The aim of this study was to explore the perceived impact and influence of cultural diversity on how neonatal and paediatric intensive care nurses care for Muslim families before and after the death of infants/children	A qualitative descriptive approach using interviews and inductive content analysis	Thirteen registered nurses participated in the study, six from the NICU and the remaining seven from the PICU	1) The cultural and religious needs of families before and after the death of an infant or child as essential to the care they provided 2) Acknowledged differences according to nationality and religion helps to support families by enabling religious practices 3) Religious practice impacted on caring of the infant/child before and after death 4) Child’s death prompted them to seek support from others and rely on their religious beliefs to cope 5) Caring and supporting each other despite their differences and learning from each other aided their coping	1) The study was conducted in one private hospital 2) The findings may be different if the sample was of mixed gender as none of the study participants were Muslim and no male nurses participated	CASP 10/10
Nascimento et al. [40] – Brazil	The objectives were to describe (1) the meaning of spirituality according to nurses working in the PICU and (2) the nurses’ experiences in providing spiritual care to children and their families	Qualitative study using face-to-face individual interviews. Data analysis used thematic analysis	Eleven PICU nurses	1) Meanings of Spirituality and Religiosity Most of the participants defined religiosity as strongly related to spirituality and as something that may be an individual belief or religion based on principles, conducts, and standards influenced by family or culture 2) Provision of Spiritual Care to Children in the PICU and Their Families 3) Nurses perceived that when they stimulate and respect the family’s faith, positive thinking, and belief in God they were also promoting serenity and reducing anxiety towards the child’s illness	1) The lack of assessment of how the participants’ religion could have influenced clinical practice and the meanings attributed to spirituality that was reported in the results 2) Some values or behaviours may predominate in the study because of snowball sampling	CASP 9/10
Neis et al. [41] – Brazil	To understand how the communication processes of the interprofessional team develop among themselves and together with the family of the hospitalised child when deciding on the adoption of palliative care in the PICU, analysing their effectiveness	Qualitative study with semi-structured interviews and content analysis	Eleven family interviews were conducted, 15 questionnaires were completed by doctors, and 20 questionnaires were completed by nurses after meetings for the palliative care decision process	1) In most of cases, there was communicative effectiveness 2) In some cases, considered ineffective, the communication process lacked feedback techniques to check for understanding and agreement of all parties on the issues addressed 3) Elements such as better psychological connection, choice of milder words, demonstration of affection, and concern for the families’ feelings lacked in some situations	N/A	CASP 10/10
Poompan et al. [48] – Thailand	To explore nurses’ perspectives on providing end-of-life care for children and their families	A qualitative descriptive approach/ Participant observation with field notes and in-depth interviews, analysed using content analysis	Twenty-five nurses	1) Assessing for entering the end-of-life stage was the initial step of end-of-life care 2) Parent involvement by HCP need to be coordinate and parents’ decision for end-of-life care should be carefully considered 3) Relieve the child’s pain and reduce the family’s grief 4) Nurses assess the needs for spiritual practices and supporting spiritual beliefs and practices 5) The nurses stated that they received inadequate policy support	The setting of the study which was in the tertiary hospital of a large city may decrease the validity of the study findings within hospitals in rural areas	CASP 10/10
Ramelet et al. [46] – Switzerland	To describe and compare characteristics of care provided at the end of life for children with chronic complex conditions and neonates who died in an ICU with those who died outside an ICU	Sub study of a nation-wide retrospective chart review. Data were extracted from child’s medical chart, community care charts or from follow-up notes in the hospital chart. Data analysis used Pearson’s chi-square test, and the Fisher exact test	149 children and neonates were included	ICU patients had more therapeutic and invasive procedures, compared with non-ICU patients. Changes in treatment plan in the last 4 weeks of life, such as do-not-resuscitate orders occurred in 40% of ICU patients and 25% of non-ICU patients	1) EOL care is defined arbitrarily for the last four weeks of life and restricted to four diagnostic groups 2) Reliability in a retrospective chart review is limited due to incomplete or missing documentation 3) This study excludes deaths within the first 24 h of life or those resulting from trauma	MMAT 7/7
Richards et al. [56]—US	To describe neonatal and paediatric critical care doctor perspectives on indicators for when and why to involve palliative care consultants	Qualitative study with semi-structured interviews and content and thematic analysis	Twenty-two ICU doctors from neonatal, paediatric, and cardiothoracic intensive care units in a single quaternary care paediatric Hospital. Fourteen from the paediatric ICU (PICU)	1) PACT provided emotional, spiritual, and informational support that helped families cope with uncertainty and navigate a complex system 2) PACT was instrumental in addressing communication needs that arose from organisational factors 3) Doctors’ paramount responsibility was to address the physiological needs of their patients 4) Each subspecialty team viewed the child through their discipline-specific perspective	Authors were unable to compare patterns between the ICUs due to having data from only one medical centre	CASP 9/10
Rubic et al. [42] – Croatia	To investigate for the first time the perceptions, experiences and challenges that healthcare professionals face when dealing with end-of-life decisions in neonatal intensive care units (NICUs) and paediatric intensive care units (PICUs) in Croatia	Qualitative study / Focused group discussion/ The constant comparative analysis method	20 doctors and 21 nurses participated in eight focus groups	1) The interrelatedness of the high emotional and cognitive demands, and the high burden associated with end-of-life issues 2) Not only physicians and nurses but families and patients also experience different emotional and decision-making burdens 3) The findings about the high variability of end-of-life procedures applied, and the various difficulties experienced during the shared decision-making process, underline the need for developing clinical and professional guidelines and the need to influence policymakers	The study from a single institution	CASP 10/10
Wu et al. [47] – Taiwan	Examined trends in mode of death, care intensity during palliative care, and resource utilisation in the PICU at the EOL	Retrospective study; Data analysis using Pearson chi-square or Fisher exact test	316 patients record whose aged less than 18 years old who died in the PICU (2011–2017)	1) A decrease in PICU mortality utilisation rate 2) A decrease in use of catecholamine infusions after do-not-resuscitate consent, in patients having limitation in life-sustaining treatment 3) An increase in withdrawal of life-sustaining treatment	1) The study’s findings are based on retrospective accounts 2) The authors were not able to examine factors that may influence parent choices such as religion and socioeconomic status 3) The study is from a single medical institution	MMAT 5/7

**Table 4:** Study characteristics

Characteristics	Number of studies (%) (*n* = 21)	Studies by
***Country***		
Australia	3 (14.3%)	[36–38]
Brazil	3 (14.3%)	[39–41]
Croatia	1 (4.7%)	[42]
Ecuador (multi-country settings)	1 (4.7%)	[43]
Saudi Arabia	1 (4.7%)	[44]
Spain	1 (4.7%)	[45]
Switzerland	1 (5%)	[46]
Taiwan	2 (9.5%)	[47]
Thailand	3 (14.3%)	[48–50]
United Kingdom	2 (9.5%)	[51, 52]
United States	4 (19%)	[53–56]
***Design and Method***		
* Qualitative study (n* = *12)*		
Constructivist grounded theory	1 (4.7%)	[36]
Participatory action research	1 (4.7%)	[50]
Qualitative descriptive study	5 (23.8%)	[41, 44, 48, 49, 56]
Qualitative study	5 (23.8%)	[38, 40, 42, 51, 52]
* Quantitative study (n* = *7)*		
Prospective case series	1 (4.7%)	[54]
Retrospective cohort study	1 (4.7%)	[39]
Prospective study	1 (4.7%)	[53]
Retrospective study	3 (14.3%)	[45–47]
Secondary analysis from trial data	1 (4.7%)	[55]
* Mixed-method study (n* = *2)*	2 (9.5%)	[37, 43]
***Participant***		
Bereaved parents	2 (9.5%)	[36, 52]
Healthcare professionals	12 (57.1%)	[37, 38, 40, 42–44, 48–51, 54, 56]
Healthcare professionals and family	1 (4.7%)	[41]
Patients	6 (28.6%)	[39, 45–47, 53, 55]

### Elements of end-of-life care in PICU

The analysis of the papers’ findings resulted in identifying three elements in end-of-life care provision for children in the PICUs: (1) Assessment of entering the end-of-life stage, (2) Parental decision-making at the end-of-life, and (3) End of life care processes consisting of care provided during the dying phase, care provided at time of death, and care provided after death. The roles of HCPs were mentioned in every stage in the care provision.

#### Assessment of entering the end-of-life stage

Three papers [48, 49, 51] raised issues on the recognition of end-of-life and concluded that a consensus is required among the HCPs involved to determine a patient entering end-of-life. Nevertheless, different approaches to end-of-life assessment and recognition were reported in each study. According to Mitchell and Dale [51] nurses were commonly the first HCP to recognise deterioration in the condition of children towards the end-of-life. Nurse participants in the study by Poompan et al. [48] conveyed that all children admitted to the PICU were assessed daily by nurses using the Palliative Performance Scale of Children (PPSC) to allow them to initiate appropriate care plans. However, nurse participants also mentioned that they could not provide care as planned because they had to wait for doctors’ decisions. In the same study, doctors used more subjective methods of evaluation when deciding on a patient’s prognosis, for example the patient’s response to treatment, likelihood of survival or minimal probability of survival. Mitchell and Dale [51] revealed that gaining HCP agreement on the recognition of end-of-life is a key obstacle to the advance care planning (ACP) process. In a study by Jongaramraung et al. [49], an end-of-life diagnosis was based on the consideration of ‘2Cs’; the Clinical symptoms of patients who failed to thrive with continued medical treatment (including deteriorating diseases), and a Consultation with other medical specialists, such as neurologists, urologists, and endocrinologists. Indeed, consultation with other medical specialists is necessary for children with complex metabolic and neurodegenerative diseases which may create challenges in recognising end-of-life [51]. The different approaches to recognising end-of-life can challenge the continuity of care and delay initiation of end-of-life care.

#### Shared decision-making with parent(s) at the end of the child’s life

Shared decision-making was considered an important element in the provision of end-of-life care. Yet no studies examining this (*n* = 14) reported any guidance or framework to underpin discussions and decision-making with parents. However, a study in Thailand by aimed to develop palliative care guidelines for doctors and nurses working in PICU in a university hospital [50]. According to this study, family meetings to discuss transitioning to end-of-life care should take place in a private area of the unit, with the conversation emphasising the child’s prognosis and the risk of adverse outcomes.

Discussions about end-of-life care decisions required interprofessional collaboration, [42] following a doctor’s confirmation of a child entering the end-of-life stage. Nurses had the responsibility to coordinate the meeting between HCPs and parents [49] and the discussion of transitioning to end-of-life care was initiated by the treating doctor [53]. Before making end-of-life care decisions, doctors discussed with parents the most appropriate options for their child. The most common options given were life-sustaining treatment (LST) withholding or withdrawing, and maximum therapeutic care [49, 53].

Several studies indicated the involvement of palliative care teams in the end-of-life care discussion. A Taiwanese study [47] concluded that incorporating a palliative care consultation service resulted in higher willingness to consent to withdrawal of LST and decreased PICU care intensity at the end-of-life. Bobillo-Perez et al. [45] examined how end-of-life care is administered when the decision is made to limit life-support in a PICU and assessed the impact of the further involvement of the Palliative Care Unit. Palliative care doctors and intensivists work together in situations where intensive care could facilitate comfort at the end of a patient’s life and enhance the quality of care. Moreover, doctors as participants in the study by Richards et al. [56] described the benefit of a Paediatric Advance Care Team (PACT) that has the capability of developing a trustworthy connection with the patient’s family, providing psychological support, and organising treatment plans that incorporate the family’s values, concerns, and point of views. This aligns with findings from Ramelet et al. [46], who conveyed that early collaboration between a specialised paediatric palliative care team and the ICU team ensure that care delivered to dying children with complex chronic conditions and their families aligns with their needs and values.

Several considerations were identified in shared decision-making with parents. In discussing end-of-life care with children and their families, both nurses and doctors have a responsibility to use effective communication [50]. In addition, HCPs perceived that parents’ comprehension of their child’s prognosis might challenge the decision-making process [52]. HCPs recognised that shared decision-making, incorporating family’s values, and goals in end-of-life decisions required parents or family members to comprehend the likely course of their child’s condition [42, 49].

#### End-of-life care processes

The elements of end-of-life care processes included care provided during the dying phase, at the time of death, and after the death of the child.

#### Care provided during the dying phase

Several common features of care for children in the dying phase were identified from nine studies, including providing comfort care [42, 48], psychosocial care to children and their families [48, 50], pain management [46, 50, 55], spiritual care [44, 48, 50, 57], continuity of care [38, 42, 50], imminent death care [50], and collecting mementoes [38].

Comfort care was interpreted differently by participants in the studies included in the review, but it was evident that in most cases it included removal of unnecessary interventions. For example, a participant in Rubic et al. [42] stated: “…to leave them on a ventilator, to turn off all inotropic support, maybe to leave some minimal infusion…” [42].

Psychosocial care for children and their families during the dying phase was specifically mentioned in two studies [48, 50]. This included regular assessments of children’s and families’ psychological reactions to illness using standard tools, documentation of information in patient charts, management of psychological problems, symptoms’ reassessment, observations for complications, and referrals to specialised HCPs as needed. Mesukko et al. [50] in their study stated that only nurses could recognise and address the psychosocial needs of children and their families during this stage. In addition, there were no guidelines on how to communicate with children about their end-of-life care, and the provision of psychosocial support relied on the understanding of individual nurses.

Some studies focused on pain management, as a core part of end-of-life care [46, 50, 55]. However, there was variation in how HCPs conceptualised, recognised and responded to pain experienced by children. It was evident that pain and other distressing symptoms were frequently disregarded, especially by doctors [50]. Although nurses appeared to be attentive to patients’ pain, by for example utilising standardised instruments, doctors were dubious of pain scores recorded by nurses and preferred to make their own conclusions about children’s pain levels. Pain medications were frequently prescribed and given to children at the end of their lives, with Ramelet et al. [46] reporting them given to 42% of dying children in the last four weeks of their life. In addition, careful consideration was given to titrating pain medication based on age and other factors to ensure personalised comfort [55].

Spiritual care was considered by healthcare professionals [44, 48, 50, 57]. Interestingly, the papers reporting on spirituality as a focus of care for children and their families in the end-of-life phase in the PICU were from Saudi Arabia [44] and Thailand [48, 50, 57]. Healthcare participants in the Mutair et al. [44] study highlighted the significance of comprehending and preparing for the religious and cultural needs of families before and following the death of a child [44]. Different approaches were reported in relation to spiritual care and in most cases HCPs considered the religion of the patient and their family to provide individualised care that would alleviate their suffering [57]. For example, involving a priest or monk to organise the ritual of making merit (Thailand) [48], and reciting Qur’an and utilising Zamzam water (Saudi Arabia) [44]. Bloomer et al. [38] point out nurses’ efforts to create normalcy amidst the sadness and grief of a child’s mortality in PICUs and NICUs such as respecting the child as a person, creating opportunities for family involvement/connection, and collecting mementoes.

#### Care provided at the time of death

Three papers reported on the care provided at the time of the child’s death. When the child dies, HCPs assist parents to say goodbye to their child [36], preserve important mementoes of the child, spend time with their child in a private setting and perform cultural and religious rituals [50]. Mutair et al. [44] specifically described certain rituals that were followed when the child dies within the Muslim context, for example orientating the child’s body towards Mecca and placing the child’s hands together to replicate praying in Islam.

#### Care provided after death

Only two papers raised matters on the care provided after the child’s death. Grunauer et al. [43] within their study incorporating 34 PICUs from 18 countries, discovered that the availability of appropriate services to support family grief and bereavement was greater in high income countries (HICs) than in low income countries (LICs). Moreover, this international multicentre study reported a statistically significant correlation between the country income level and the availability and quality of grief and bereavement care (GBC) for PICU patients, their families, and HCPs, meaning that the higher the income of the country, the higher the provision of GBC.

Mesukko et al. [50] described components that should be considered in providing bereavement care for parents/family members and healthcare professionals. This included helping parents come to terms with the reality of their loss, offering condolences to grieving parents or family members by attending memorial services and connecting families with other parents who have encountered a similar loss, self-help organisations, or professional counselling or bereavement services. The authors also suggested bereavement care for healthcare professionals, including peer support, group debriefings, psychological and spiritual counselling, and educational programs [50].

## Discussion

**Fig. 2:**
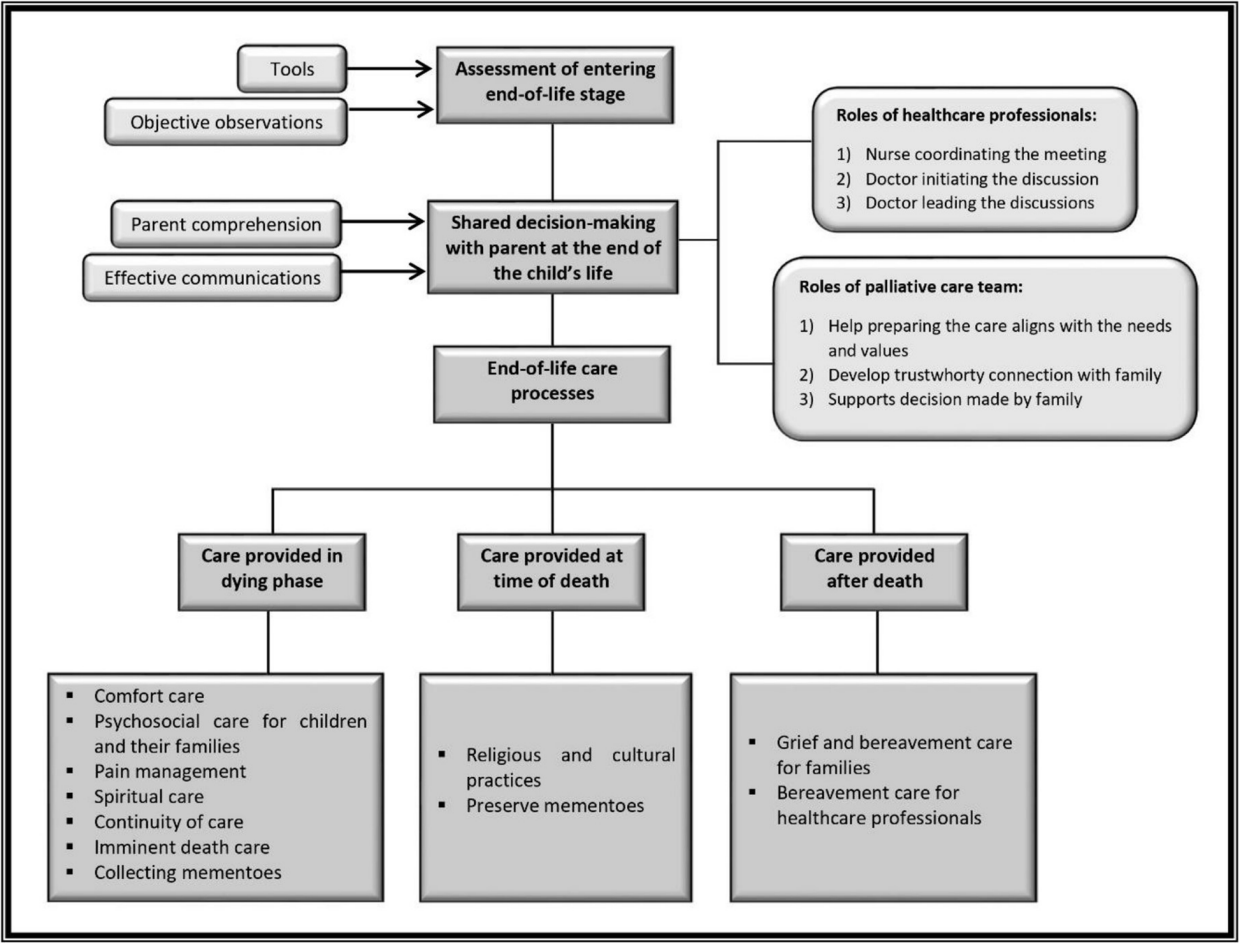
Overview end-of-life care provision in paediatric intensive care unit

This review aimed to identify and synthesise literature related to the essential elements in the provision of end-of-life care in the PICU from the perspectives of both healthcare providers and families PICU. Figure 2 presents a diagrammatic representation of the findings of this review.

The papers included in this review represented PICU end-of-life care worldwide. Although end-of-life care in the PICU may have been explored in a different manner within the different countries, a shared understanding of the value of quality end-of-life care in the PICU by considering family involvement and needs was evident. Nonetheless, one of the findings indicated that HICs had more access to adequate resources to provide optimal care than LICs [43]. Therefore, it is essential to support the conduct of paediatric palliative care research in low middle-income countries (LMICs), since the implementation of end-of-life care is still in its infancy [58], to develop effective and efficient country specific end-of-life models of care.

It was evident that different healthcare professionals and religious representatives were involved in the care of dying children and their families, including varying palliative care approaches between countries [42, 45, 47, 50, 52]. The difference in palliative and end-of-life care approaches found in this review are consistent with the palliative care models described by Nelson et al. [59], who conveyed that there are two main models of palliative care in intensive care units, namely the consultative model, where PICU HCPs request the involvement of palliative care practitioners when they feel it is needed, and the integrative model, which embeds palliative care principles into daily practice. The use of any model of PICU palliative care can only enhance the care provided to dying children and their families. Indeed, well organised palliative care can reduce ICU length of stay, non-beneficial treatments, conflict over care goals, family depression, and increase patient comfort and family satisfaction [59].

The first element in the end-of-life care provision in the PICU identified in this review is the recognition of entering the end-of-life stage. World Health Organization [60] stated that recognising the disease trajectory is one of the essential competencies for HCPs in paediatric palliative care. Although different approaches were reported, one common aspect was that HCPs discussed their views before reaching an agreement [48, 49, 51]. It was also recognised that it is challenging to recognise when a child is dying. According to Australian Commission on Safety and Quality in Health Care [61], predicting prognosis and precise time of death can be challenging, as children die from a variety of illnesses, often from ailments that are less common among adults. Moreover, a wide range of illness trajectories can result from the diversity of conditions. Predicting death is challenging, even when the child is recognised to have a potentially life-shortening condition [62]. For this reason, many advocate advance care planning for children recognised to have a life-limiting or life-threatening condition, which can plan for multiple scenarios, including emergency events, deteriorations, and end-of-life [63].

Only one study [48] mentioned the use of a tool called PPSC by nurses to assess whether a paediatric patient is entering the end-on-life phase and requiring end-of-life care. However, there is no detailed information regarding this instrument in the study. Since it is difficult to predict dying and exact time of death, it is advisable to use tools to assess patients’ palliative care needs and evaluate the effectiveness of palliative care provided. One such instrument is the Paediatric Palliative Screening Scale (PaPaS Scale) developed by Bergstraesser et al. [64]. A study by Al-Gharib et al. [65] has modified The Needs at End of Life Screening Tool (NEST) questionnaire to assess the effectiveness of palliative care given to children. Incorporating palliative care needs assessment tools into routine care enables people to receive the right care when they most need it from the people or service that is most appropriate to address their needs [66]. According to Australian Commission on Safety and Quality in Health Care [61], regular use of modest trigger instruments and questions can encourage doctors to utilise their clinical judgement to decide whether a child could gain benefit from end-of-life care. In addition, it is essential to acknowledge when a child is approaching the end of their life in order to provide appropriate, compassionate, and timely end-of-life care, [61].

Findings indicated that different professionals have different levels of recognition and views about end-of-life care. For example, nurses tend to recognise end-of-life signs first [48], and doctors tend to disregard information about pain [50]. Achieving consensus on treatments and care plans is challenging if people have different understandings and beliefs which can also hinder shared decision-making with parents [50]. Additionally, parents and HCPs can have disagreements in the decision of the end-of-life care for patients if parents were unable to choose to withdraw care or limit the intervention [67]. Hence, collaborative working between HCPs is needed in discussions with parents for decision-making, particularly to convey medical uncertainty to the patient’s parents. Meetings between parents and doctors to discuss transition to end-of-life care can be coordinated by nurses [48], with doctors initiating and leading the discussion [38, 42]. In these discussions, which should take place in appropriate rooms that offer privacy [50], any HCPs involved must ensure that parents thoroughly understand their child’s complex condition and the treatment options by utilising effective communication skills [41]. Shared decision-making requires that parents who act as their child’s surrogate decision-makers and health care professionals collaborate to reach decisions that consider family preferences and medical evidence [68]. Shared decision-making provides several advantages for patients, families, and HCPs, including enhanced patient or family comprehension, decreased decisional conflict, improved participation and engagement in care, enhanced coping mechanism, and more effective healthcare resources utilisation [68].

Early collaboration between the paediatric palliative care team and the PICU team appears to facilitate care for dying patients and families in meeting their needs, assist in developing a trusting relationship with the patient’s family, and organise a treatment plan that involves the family [46, 56]. Therefore, a further study is recommended to evaluate the best approach to integrating the palliative care team in the PICU, which may contribute to improving the delivery of end-of-life care. In line with a previous review [18], it may be that an integrated approach is necessary for effectively transitioning patients in the PICU from LSTs to end-of-life care.

During the three stages of end-of-life care processes (dying phase, time of death, and after the death) as identified in this review, it was evident that comfort and dignity were ultimate goals. Apart from management of distressing symptoms and psychological support, spiritual care was also used to achieve these goals. Spiritual care was more evident in countries where religion plays an important role for example in Saudi Arabia [44] and Thailand [48, 50]. According to Pravin [69], spirituality offers numerous benefits to families, including providing solace during difficult decisions, comfort and validation to bereaved families by the use of religion, and spirituality also helps parents hold onto hope and maintain a spiritual connection with a deceased child. Collecting and preserving mementoes between parent and child are also considered to be important during the dying phase and the time of the child’s death [38, 50]. Clarke and Connolly [70] described that memory-making and tangible mementoes had an enormously favourable effect on parents, especially in dealing with loss and grief. Therefore, it is crucial for HCPs to be able to recognise, assess, and address patients’ and their families’ spiritual needs to enhance the quality of end-of-life care [71].

### Strengths and limitations

This integrative review explored the elements of end-of-life care provision in PICU based on papers that were retrieved through a comprehensive and systematic search. Including 21 studies from 11 different countries demonstrates that findings could be relevant globally. In addition, the use of PRISMA framework ensured transparent and comprehensive reporting. Most of the review processes were undertaken independently by two reviewers including the study selection and data extraction. The evaluation of the included studies and data synthesis were conducted by one reviewer and verified and validated by all reviewers. This approach appears to be effective in terms of time while still ensuring the rigour and validity of the process. Furthermore, all included papers were of good quality and the findings are supported by existing evidence, giving confidence in the final conclusions. Despite a comprehensive search strategy, it is acknowledged that not all journals are listed in the databases, so literature may have been missed. However, hand searches of included papers and grey literature searches should have minimised this risk. Including papers only in English language may have resulted in missing potentially relevant information in other languages.

## Conclusion

This review identified several elements of delivering end-of-life care in the PICU from the perspectives of HCPs and families. The focus of care provided can differ at each stage depending on HCPs’ and families’ preferences, specifically during the dying phase, at the time of death, and after the child died. This highlights the importance of tailoring end-of-life care to individual needs, beliefs and rituals. This review emphasises the importance of HCPs’ collaboration to provide optimum end-of-life care in the PICU. In addition, this review reveals that early involvement of the palliative care team in end-of-life care in the PICU can be beneficial.

Based on the findings of this review, future research should focus on identifying effective approaches to recognise children entering the end-of-life stages and exploring how best to assess and address end-of-life care needs of patients and their parents. The education and training of both currently practicing and future PICU HCPs can also be the focus of future research since it is conceivable that specific resources and training dedicated to palliative care will have a significant impact on end-of-life practices in PICUs. It is evident that end-of-life care in PICUs is influenced by cultural and socioeconomic factors, hence the development of palliative and end-of-life care models should take these factors into account. The findings of this review provide some common PICU end-of-life elements that could be adopted in the development of country or even individual PICU specific end-of-life care models.

## Data Availability

No datasets were generated or analysed during the current study.

## Abbreviations

PICUs: Paediatric Intensive Care Units
HCPs: Healthcare Professionals
EMBASE: Excerpta Medica dataBASE
CINAHL: Cumulative Index of Nursing and Allied Health Literature
MEDLINE: Medical Literature Analysis and Retrieval System Online
PsycINFO: Psychological Information Database
EthOS: Electronic Theses Online Service
UK: United Kingdom
US: United States
PRISMA: Preferred Reporting Items for Systematic Reviews and Meta-Analyses
PICOTS: Population, Intervention/Interest, Comparison/Context, Outcome, Time, and Study Design
NICU: Neonatal Intensive Care Unit
CASP: Critical Appraisal Skills Programme
MMAT: Mixed Methods Appraisal Tool
PPSC: Palliative Performance Scale of Children
LST: Laife-Sustaining Treatment
PACT: Paediatric Advanced Care Team
HICs: High Income Countries
LICs: Low Income Countries
GBC: Grief and Bereavement Care
LMICs: Low Middle-Income Countries
PaPaS Scale: Paediatric Palliative Screening Scale
NEST: Needs at End of Life Screening Tool
UMICs: Upper Middle-Income Countries
